# PREnatal Syphilis point-of-care ScreenING in an urban low-barrier clinic (PRESSING) study: Protocol for a prospective single arm feasibility trial

**DOI:** 10.1371/journal.pone.0360150

**Published:** 2026-10-22

**Authors:** Njideka Sanya, Mary-Elizabeth Vaccaro, Medora Uppal, Sarah Glen, Chelsea Kirkby, Tehreem Zafar, Janet Gasparelli, Kate MacNeil, Stephanie Milliken, Jeffrey Pernica, Jacqueline Wong, Donald Brody Duncan, Marek Smieja, Amanda Ramdyal, Robin Lennox, Tim O’Shea, Anna Cvetkovic, Lawrence Mbuagbaw, Suzanne Turner, Laura Erdman

**Affiliations:** 1 Department of Pediatrics, McMaster University, Hamilton, Ontario, Canada; 2 Department of Social Work, McMaster University, Hamilton, Ontario, Canada; 3 YWCA Hamilton, Hamilton, Ontario, Canada; 4 De dwa da dehs nye>s Aboriginal Health Centre, Hamilton, Ontario, Canada; 5 Department of Pathology and Molecular Medicine, McMaster University, Hamilton, Ontario, Canada; 6 Department of Family Medicine, McMaster University, Hamilton, Ontario, Canada; 7 Department of Medicine, McMaster University, Hamilton, Ontario, Canada; 8 Department of Health Research Methods, Evidence, and Impact, McMaster University, Hamilton, Ontario, Canada; Public Library of Science, UNITED KINGDOM OF GREAT BRITAIN AND NORTHERN IRELAND

## Abstract

**Introduction:**

Syphilis has resurged across North America, including syphilis in pregnancy. Congenital syphilis can be prevented by timely treatment of syphilis in pregnancy, but conventional syphilis screening is problematic for people facing barriers to care. Syphilis point-of-care tests (POCT) have improved prenatal syphilis management in low- and middle-income countries. There is minimal literature regarding POCT feasibility and effectiveness for prenatal syphilis screening in high-income countries, where barriers and challenges may differ.

**Methods:**

Academic and community partners co-designed a prospective single-arm feasibility trial to evaluate syphilis POCT implementation at a low-barrier prenatal clinic in Hamilton, Canada. The INSTI HIV/syphilis POCT was integrated into routine clinical care for pregnant people with ≥1 risk factor for syphilis (unstable housing in the past year; substance use or accessing addictions services in the past year; > 1 sexual partner in the past year; history of sexually transmitted or blood-borne infection; presentation to care >20 weeks gestation). Sexual partners of newly diagnosed pregnant people are also offered POCT. Individuals offered POCT are invited to enroll in the study. The primary feasibility outcome is the proportion of pregnant participants who accept POCT at first offer. Secondary feasibility outcomes include valid POCT results on clinician’s first attempt and accurate POCT interpretation by clinicians. Secondary clinical outcomes include time to treatment initiation and completion of treatment among syphilis-positive participants. To promote inclusion of groups facing structural barriers to research, individuals declining study participation are enrolled for limited prospective chart review (primary feasibility outcome only). Target enrollment is N = 62 pregnant participants over 18 months. Baseline participant characteristics and outcomes will be descriptively analyzed. Community-based knowledge mobilization is ongoing.

**Conclusion:**

This pilot study will generate novel feasibility data regarding prenatal syphilis screening with POCT in high-income countries and inform design of a multicenter implementation trial.

**Trial registration:**

Clinicaltrials.gov NCT06829602; February 17, 2025. https://clinicaltrials.gov/study/NCT06829602

## Introduction

Syphilis has dramatically increased in North America over the past decade, primarily driven by the social determinants of health [1]. Females of child-bearing age are disproportionately affected [2–5], thus posing risks for syphilis in pregnancy and adverse outcomes such as miscarriage, stillbirth, premature delivery, and congenital syphilis [6–11]. In Canada, rates of infectious syphilis in females of child-bearing age increased from 2.3 per 100,000 in 2014 to 53.8 per 100,000 in 2023, and confirmed early congenital syphilis cases rose from 1 to 53 cases [2,3]. In the United States, rates of primary and secondary syphilis among females of child-bearing age increased by 676% between 2012 and 2021, and the number of congenital syphilis cases rose from 335 to 2865 [12]. Congenital syphilis can cause severe or fatal infection in infants [9–11] and long-term neurological and musculoskeletal sequelae [7]. It has also been linked to excess mortality in early childhood [13].

Congenital syphilis is almost always preventable by timely diagnosis and treatment of syphilis in pregnancy, and concurrent treatment of sexual partners to avoid maternal re-infection [14–16]. Treatment effectiveness is highest prior to third trimester [14]. Canadian guidelines recommend universal screening at the first prenatal visit and repeat screening at 28–32 weeks and delivery (or more frequently) for people at higher risk of infection [17]. U.S. guidelines now recommend universal screening at all three timepoints [18].

Syphilis screening may be challenging for populations most affected by this infection. Syphilis in pregnancy is associated with low income, substance use, mental health conditions, housing instability, and rural and remote residence [19–22]. These social determinants of health pose barriers to prenatal care related to transportation, language, stigma, mistrust of healthcare systems, and concerns about loss of child custody [22–27]. Cases of congenital syphilis are associated with delayed or absent prenatal syphilis testing and/or treatment of syphilis in pregnancy [12,25,28,29].

The conventional syphilis serological test itself has several problematic features for populations facing barriers to care. Firstly, it requires a venous blood sample. People who use intravenous drugs are at risk of unsuccessful venipuncture [30], and this can be a barrier to prenatal syphilis screening. Secondly, turn-around time for syphilis serology is often days to weeks [27,31]. By the time test results are available, various barriers may impede communication of results and follow up for treatment [22,27,32]. These diagnostic issues create inequitable gaps in syphilis care, with potential for adverse pregnancy and neonatal outcomes.

Syphilis point-of-care tests (POCT) may help facilitate prenatal syphilis screening and treatment in this context [27,33,34]. These rapid immunoassays use a finger-prick sample to detect treponemal and/or treponemal antibodies in blood, often in combination with an HIV test, and produce results within minutes. Compared to laboratory-based serological assays, POCTs have excellent specificity and good sensitivity [35–37]. HIV/Syphilis POCTs have been widely implemented in prenatal care in low- and middle-income countries, given geographic and resource-related barriers to care and limited laboratory facilities. In these settings, POCTs are highly acceptable to pregnant people and clinicians, and increase syphilis screening rates, same-day treatment, and partner treatment [38,39]. Modelling suggests that syphilis POCTs reduce adverse perinatal outcomes and are cost-effective, even in low-prevalence settings [40–43]. Challenges with implementation include insufficient staff training and time; inadequate quality assurance; POCT cost and availability; and pregnant people declining POCT due to lack of understanding of syphilis or fear of a positive result [39,44,45].

In high-income countries, syphilis POCT screening has been recommended for pregnant people facing barriers to care [26,27] – but limited evidence supports this recommendation, and this is a noted research gap [46]. In non-pregnant adults at risk of syphilis in high-income countries, syphilis POCTs are acceptable and facilitate same-day treatment [47–53]. However, it is unclear if these findings, or the success of prenatal syphilis POCT in low- and middle-incomes countries, can be extrapolated to marginalized pregnant people in high-income countries. These individuals and their healthcare providers face a confluence of competing priorities related to basic needs (e.g., food insecurity, unstable housing), prenatal care, and complex medical issues such as substance use management and mental health concerns. Early results are promising: an implementation study at an Emergency Department in Texas found that opt-out syphilis POCT for pregnant people with limited prenatal care increased syphilis screening from 2% to 56.4%. 80% agreed to syphilis POCT, 100% found POCT acceptable, and the 4 patients who tested positive received same-day treatment [31]. Similarly, POCT implementation in a Chicago Emergency Department found increased rates of syphilis screening and increased diagnoses of syphilis in pregnant people [54]. Additional studies are needed to replicate these findings in different prenatal care settings and healthcare systems. Evaluating the impact of syphilis POCT on treatment of pregnant people and their sexual partners would be valuable, since partner management is often suboptimal [55,56].

Below we describe the protocol for a pilot feasibility trial of syphilis POCT implementation in a low-barrier prenatal clinic in urban Canada. Results will inform the design of a larger implementation trial to guide clinical practice and public health policy. Given the social inequities associated with syphilis and the historical harms of syphilis research [57–59], this project has been co-developed with community partners to prioritize psychological and cultural safety for research participants, ensure benefit for communities affected by syphilis, and guide knowledge mobilization. Partners include people with lived experience of syphilis in pregnancy and/or substance use in pregnancy; the YWCA Hamilton, a social service organization that supports people with substance use and homelessness [60]; De dwa da dehs nye > s Aboriginal Health Centre, which provides low-barrier healthcare services to urban Indigenous communities [61]; and Indigenous consulting group Niswi Kwe [62]. Indigenous communities in Canada are disproportionately affected by syphilis and congenital syphilis [19,20,63], related to intergenerational trauma and systemic barriers to care due to legacies of colonization and pervasive racism [64,65]. Indigenous Peoples are strong sovereign nations with inherent rights, including the right to make decisions about their lives and issues affecting their communities, and are partners in the response to the syphilis epidemic.

## Materials and methods

### Study aims

The primary feasibility aim of this study is to measure acceptability of HIV/syphilis POCT among pregnant people with pre-defined risk factors for syphilis. This outcome is person-centered and has direct relevance for POCT feasibility and design of a multi-site trial. Secondary aims are to evaluate clinician performance of POCT in the clinical environment; to establish baseline data regarding timeliness and adequacy of treatment of syphilis in pregnancy at the study site; and to evaluate whether POCT expedites treatment of syphilis-positive pregnant people and their sexual partners.

### Study design

The PRESSING study is a prospective single arm feasibility trial with elements of chart review methodology. The study protocol is reported in accordance with the Standard Protocol Items: Recommendations for Intervention Trials guidelines (SPIRIT). See Fig 1 for the schedule of enrollment, interventions, and assessments, and S1 File for the SPIRIT Checklist.

**Fig 1:**
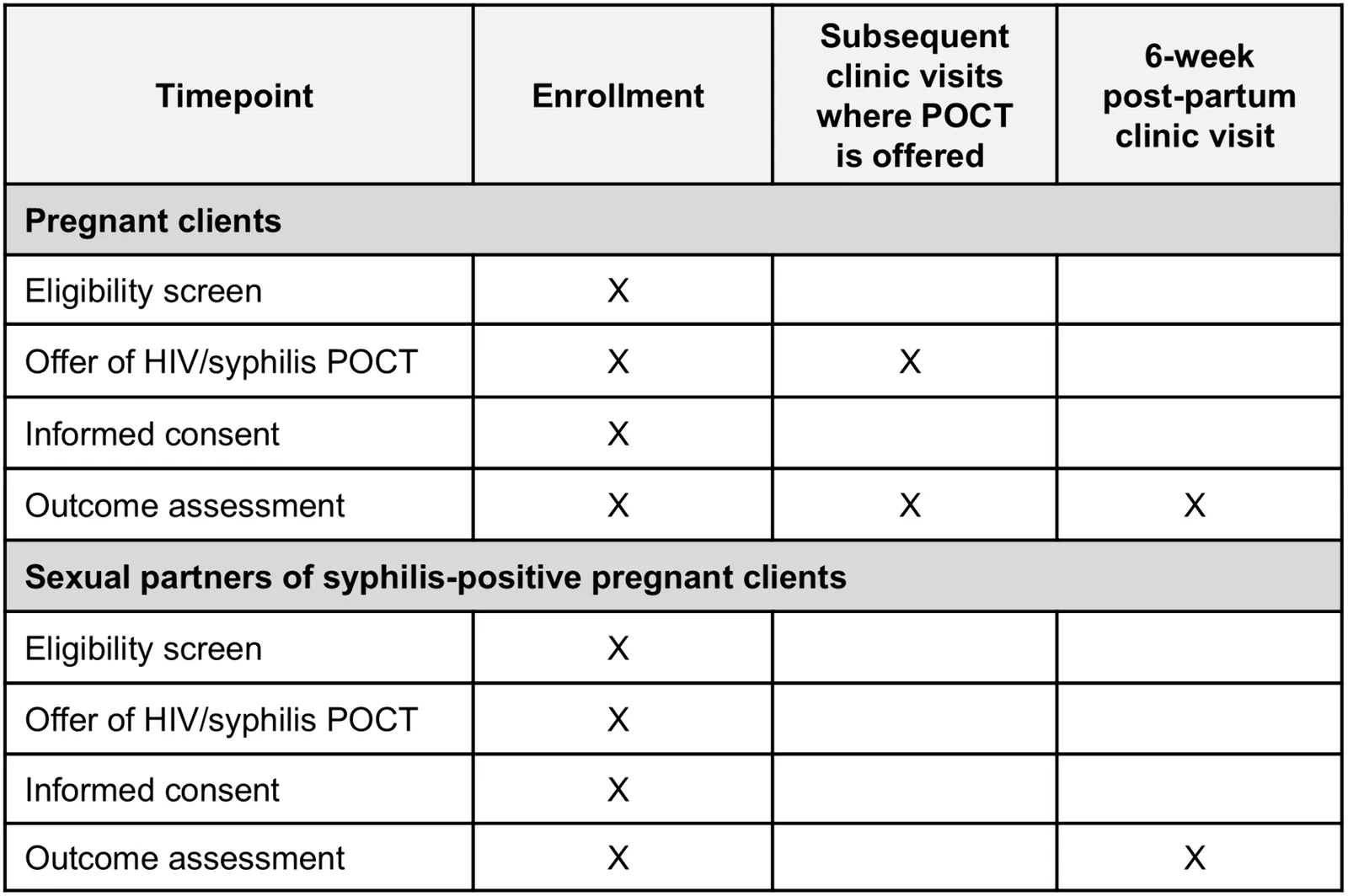
SPIRIT participant timeline: Schedule of enrolment, interventions, and assessments.

The Maternity Centre of Hamilton (MCH) is a low-barrier prenatal clinic in downtown Hamilton, Ontario. MCH recently implemented a Health Canada-approved HIV/syphilis POCT [66] into routine clinical care for pregnant people with pre-defined risk factors for syphilis, and for sexual partners of syphilis-positive clients. POCT implementation will be prospectively evaluated over 18 months (June 18, 2025 to December 18, 2026), with chart review integrated in the following two ways (Fig 2):

1. Prospective chart review: This study was designed with awareness of barriers to research participation among people disproportionately affected by syphilis. For example, people who use substances may decline research involvement due to stigma, mistrust of researchers’ intentions, and fear of authorities [67–69]. Indigenous community members may decline due to historical research atrocities [70–72]. Yet, it is critical to include these voices. For pregnant people who decline study enrollment, the primary feasibility outcome (POCT acceptance at first offer) is collected via prospective chart review, with waived consent. Supported by community partners, this mechanism is intended to promote representativeness of the study sample with minimal risk to participants.
2. Retrospective chart review: To estimate the effect of POCT implementation on the timeliness and adequacy of treatment of syphilis in pregnancy, we will perform a retrospective chart review to establish baseline data over the previous 3 years at MCH. We felt it was unethical to have a control group with delayed access to POCT in the prospective study, given the importance of timely syphilis treatment in pregnancy and barriers to follow-up among MCH clients.

**Fig 2:**
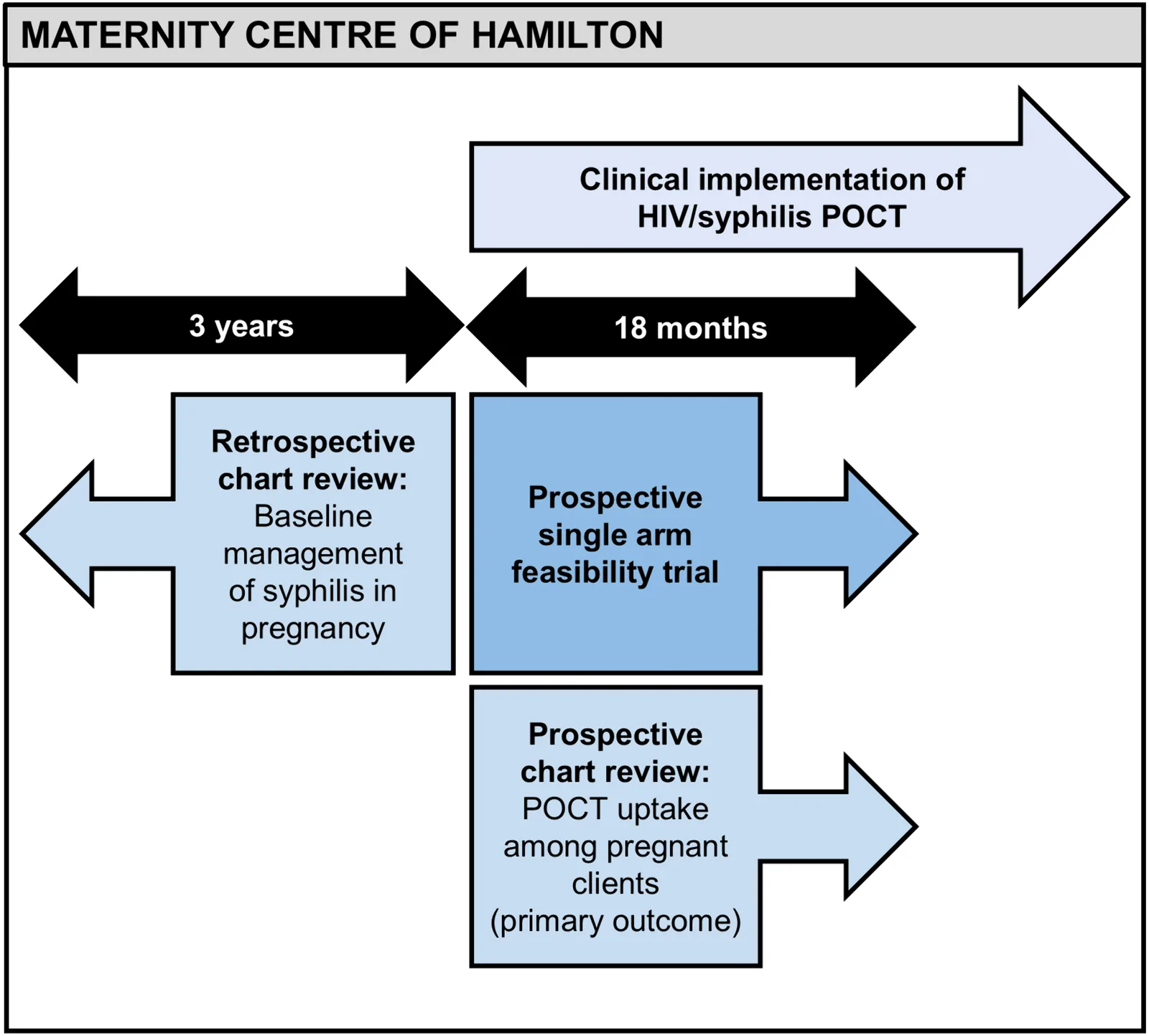
PRESSING study design. POCT: Point-of-care test.

### Study setting

MCH is a community-based prenatal clinic affiliated with McMaster University’s Department of Family Medicine, designed to serve urban populations facing barriers to prenatal care such as low income, substance use, housing instability, and newcomer status [30]. Care is delivered by family physicians, nurse practitioners, and midwives. MCH offers self-referral, walk-in visits, and embedded social services. MCH runs a weekly specialized clinic called the Program for Substance Use in Pregnancy (PROSPR) for integrated prenatal and substance use care, with a focus on harm reduction. Reproductive health outreach workers from YWCA Hamilton are integrated into PROSPR: they engage pregnant people in shelters and street-based settings, and provide support to access care and follow up.

In 2023, MCH clinicians noted an anecdotal increase in syphilis in pregnancy cases, especially among people using substances. They identified barriers to diagnosis and treatment that syphilis POCT may help address, including challenges with venipuncture and follow up.

### Study population

Two populations are under study:

1. Pregnant people receiving prenatal care at MCH with pre-defined risk factors for syphilis (based on syphilis risk factors identified in the Canadian literature and Canadian syphilis guidelines [19,20,32,73]).
2. Sexual partners of syphilis-positive pregnant people.

Study inclusion and exclusion criteria are displayed in Table 1. For ease of study implementation, they were designed to be identical to clinical criteria for offering POCT.

**Table 1:** Inclusion and exclusion criteria for the two study populations.

	Inclusion criteria	Exclusion criteria
Pregnant people	1. Pregnant clients at MCH^a^ with at least one of the following risk factors for syphilis, as identified by MCH clinicians: i. Substance use^b^ or accessing addiction services in the past year; ii. Unstable housing^c^ in the past year; iii. Multiple sexual partners in the past year; iv. History of STBBI^d^; **OR** v. Late to prenatal care (>20 weeks gestation)^e^ **AND** 2. Eligible for syphilis screening at that visit, per Public Health guidelines [17]: i. First prenatal visit; ii. Repeat syphilis screening at 28–32 weeks if ongoing risk of syphilis infection; iii. Additional screening at clinician’s discretion if ongoing risk of syphilis infection; **OR** iv. No prior syphilis screening.	Previous diagnosis of syphilis, by verbal report and/or chart review (since POCT cannot distinguish between active syphilis and syphilis that has been adequately treated); **OR** Deemed to lack capacity to consent to study participation (e.g., intoxicated).
Partners of syphilis-positive pregnant people	Sexual partners of pregnant clients who are newly diagnosed with syphilis in pregnancy, by POCT and/or laboratory-based syphilis serology; **AND** Physically present in MCH clinic.	Exclusion criteria as above.

a HIV: Human Immunodeficiency Virus; MCH: Maternity Center of Hamilton; POCT: point-of-care test; STBBI: Sexually transmitted and blood borne infections;

b Problematic alcohol or cannabis use, non-prescribed opioid use, cocaine use, methamphetamine use, or intravenous drug use.

c Homeless/houseless, street-involved, shelter use, staying with friends/family, or facing eviction.

d Diagnosed with HIV, Hepatitis C, Hepatitis B, gonorrhea, chlamydia, genital herpes simplex, or trichomonas, as per client’s report and/or medical records.

e Defined by clinician consensus; refers to prenatal care at MCH or elsewhere.

### Study procedures

Study procedures are illustrated in Fig 3, in relation to clinical care. As part of routine clinical care at MCH, clinicians offer HIV/syphilis POCT to pregnant people meeting clinical criteria (identical to study inclusion and exclusion criteria, above) each time they qualify for laboratory-based syphilis serological screening as per Canadian guidelines [73]. If a pregnant person tests positive, they are immediately treated and any eligible sexual partners present in clinic are offered HIV/syphilis POCT and laboratory-based syphilis serology.

**Fig 3:**
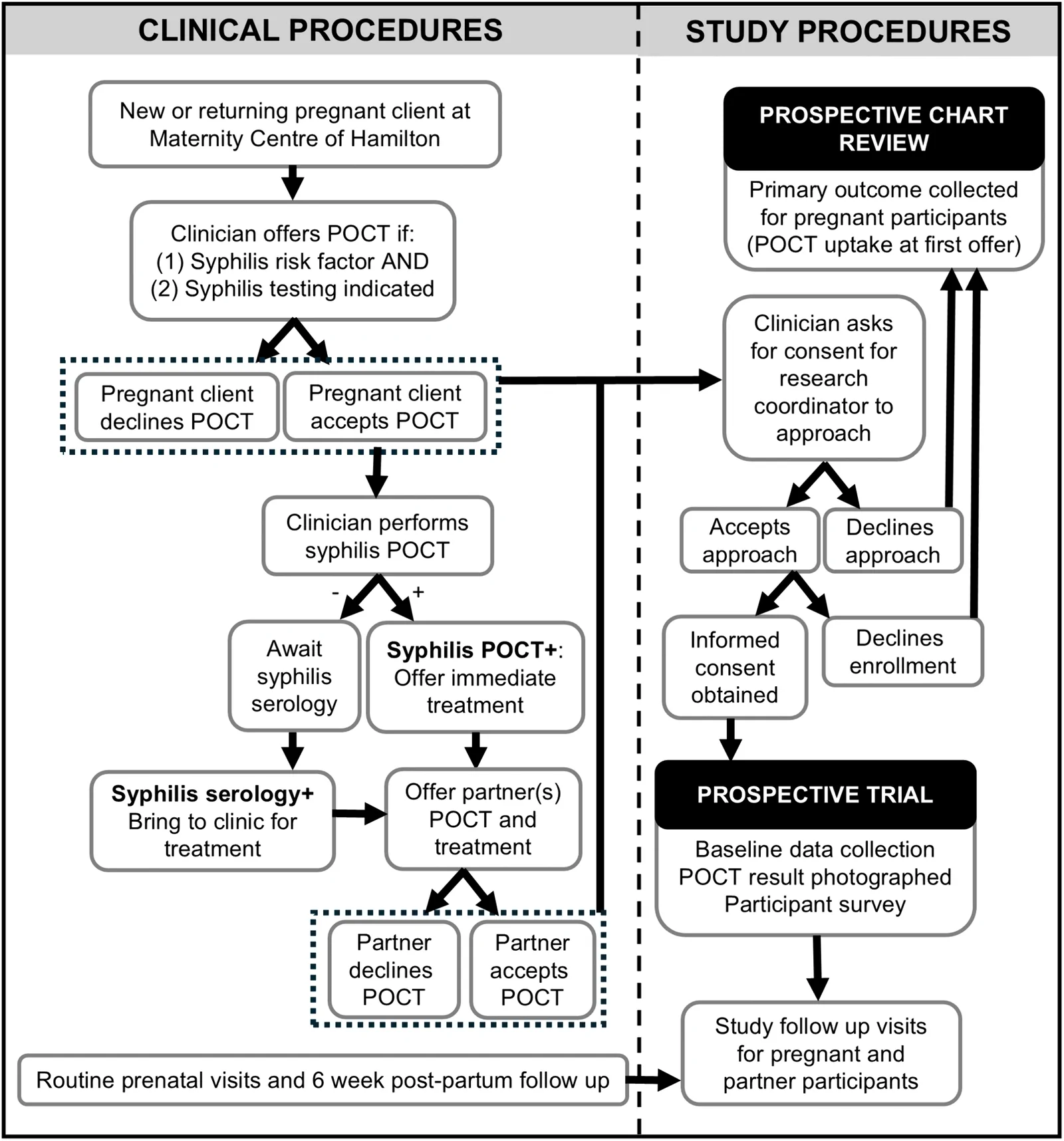
Study procedures. POCT: Point-of-care test.

After a pregnant person or sexual partner is offered POCT, and regardless of their response, the clinician introduces the study. If they consent to approach, research personnel provides information about the study and seeks informed consent. The consent discussion explicitly highlights that data collection includes sensitive questions about substance use history, sexual history, and race/ethnicity, to understand how different groups perceive POCT and with the goal of equitable implementation and universal benefit.

Participants have the option of providing written or verbal informed consent (with the approval of our research ethics board). Verbal consent was incorporated given expected low literacy and potential language barriers in the study population, which affect the ability to read and understand a written consent form. Moreover, given the systematic marginalization experienced by identified populations, the requirement for a written signature may dissuade some people from participating. Research personnel review the two consent options with eligible participants, who determine which option is most suitable for their circumstances. For verbal consent, the consent form is read aloud to the eligible participant in the presence of a witness, with language interpretation as required. The research personnel and witness sign the consent form to attest that informed verbal consent was provided. For 13–15-year-olds who are eligible for the study, the ethics committee waived the requirement for parental consent, as they have demonstrated mature decision-making capacity in continuing with their pregnancy.

The following baseline data are collected: feedback on the POCT experience or why participants declined POCT; self-reported gender and race/ethnicity (according to the Canadian Institute of Health Information race-based and Indigenous identity data standards) [74]; migration in the past 5 years; gestational age, gestational age at first prenatal visit, and pregnancy complications (for pregnant participants); substance use and addiction services; housing situation; sexual partners and STBBI history; syphilis symptoms; previous syphilis testing; whether POCT completed (with reasons for declining, if given); POCT process and results; and syphilis management if POCT positive (staging, treatment, partner management). The POCT result is photographed and uploaded to a secure server, for evaluation of test interpretation by clinicians.

Pregnant participants are followed up at every subsequent clinic visit that they are offered POCT (Fig 3), and all participants are followed up at the 6-week post-partum visit. The following data are collected at each follow-up: updates on pregnancy complications (for pregnant participants only); updates on syphilis risk factors, symptoms, testing, and management; and syphilis testing and treatment at the current visit. If participants do not attend scheduled follow-ups, and three attempts to contact them over the phone are unsuccessful, research personnel review MCH medical records to obtain data (as outlined in the consent form).

If a pregnant person declines approach by research personnel or declines study participation, prospective chart review is conducted to document the date that POCT was offered, and whether POCT was accepted or declined (Fig 3).

A retrospective chart review over the 3 years prior to POCT implementation is underway to generate baseline data regarding management of newly diagnosed cases of syphilis in pregnancy. Cases will be identified by a positive syphilis serology result and/or a documented syphilis diagnosis. The following data are collected: age; self-reported gender and race/ethnicity; migration in the past 5 years; gestational age at first prenatal visit and pregnancy complications; substance use and addiction services; housing situation; sexual partners and STBBI history; and syphilis symptoms, testing, and management (staging, treatment, follow up testing, partner management).

### Outcomes

Outcomes are displayed in Table 2. The primary feasibility outcome is the proportion of pregnant participants who accept POCT the first time it is offered. We defined the feasibility threshold at 80%, based on acceptability of syphilis POCT in prenatal care in other studies [31,38] and acceptability of HIV and Hepatitis C POCT in non-pregnant populations [75–77].

**Table 2:** Study outcomes and planned methods of analysis^a^.

	Outcome	Planned analysis	Feasibility threshold
Acceptability of POCT	**Primary feasibility outcome**		
	1. Proportion of pregnant participants who undergo POCT^b^ the first time it is offered	n/N (%), 95% CI	**>80%**
	**Secondary feasibility outcomes**		
	2. Proportion of pregnant participants who undergo POCT at least once during pregnancy	n/N (%), 95% CI	**>80%**
	3. Proportion of pregnant participants who undergo POCT and report willingness to undergo POCT in future, based on the post-POCT survey	n/N (%), 95% CI	**>80%**
	4. Proportion of partner participants who undergo POCT and report willingness to undergo POCT in future, based on the post-POCT survey	n/N (%), 95% CI	**>80%**
POCT performance by clinicians	5. Proportion of POCTs that produce a valid result on the clinician’s first attempt	n/N (%), 95% CI	**>90%**
	6. Proportion of POCT results correctly interpreted by clinicians^c^	n/N (%), 95% CI	**>90%**
POCT expediting treatment for **pregnant** participants	**Secondary exploratory clinical outcomes**		
	7. Proportion of syphilis POCT-positive pregnant participants who initiate penicillin treatment on the same day	n/N (%), 95% CI	N/A
	8. Time from the first positive syphilis test to communication of test results to syphilis serology-positive pregnant participants	Median (IQR) or mean (SD)	N/A
	9. Time from the first positive syphilis test to first penicillin treatment among syphilis serology-positive pregnant participants	Median (IQR) or mean (SD)	N/A
	10. Proportion of syphilis serology-positive pregnant participants who receive any syphilis treatment during pregnancy	n/N (%), 95% CI	N/A
	11. Proportion of syphilis serology-positive pregnant participants who receive recommended treatment during pregnancy	n/N (%), 95% CI	N/A
POCT expediting treatment for **partner** participants	12. Time from the pregnant participant’s first positive syphilis test to first penicillin treatment of the partner participant	Median (IQR) or mean (SD)	N/A
	13. Proportion of partner participants who receive any syphilis treatment during the pregnancy	n/N (%), 95% CI	N/A
	14. Proportion of partner participants who receive recommended syphilis treatment during the pregnancy	n/N (%), 95% CI	N/A

a Analyses will be descriptive. Binary outcomes will be reported as n/N (%) with two-sided 95% confidence intervals. Continuous time outcomes will be summarized using median (IQR) when skewed or mean (SD) when normally distributed. No formal hypothesis testing is planned.

b CI, confidence interval; IQR, interquartile range; POCT, point-of-care test; SD, standard deviation.

c Per consensus of 2 blinded independent study staff reviewing digital photographs of result.

Secondary feasibility outcomes assess other dimensions of POCT acceptability and test performance by clinicians, with feasibility thresholds as indicated. Secondary exploratory clinical outcomes measure POCT effectiveness for expediting syphilis treatment of pregnant and partner participants.

### Sample size determination

If the primary feasibility outcome (POCT acceptance by pregnant people on first offer) is 80%, and to ensure that the width of the 95% confidence interval around this estimate is at most 10%, at least 62 pregnant people should be offered POCT over the study period.

### Statistical analysis

Descriptive statistics will be used to analyse baseline characteristics of pregnant participants, syphilis-positive pregnant participants, and partner participants. Binary variables (e.g., self-reported gender, substance use) will be reported as counts and proportions with 95% confidence intervals. Continuous variables (e.g., age) will be presented as median with first to third quartiles (Q1,Q3) when skewed, or mean with standard deviation (SD) when approximately normally distributed.

Feasibility outcomes will be analysed as n (%) with 95% confidence intervals and interpreted against pre-specified thresholds.

For exploratory clinical outcomes related to management of syphilis infection in pregnancy, we will compare participants diagnosed with syphilis by POCT, versus those diagnosed by laboratory-based syphilis serology (i.e., if declined POCT, or if POCT was negative). Categorical outcomes will be compared using Chi-square test or Fisher’s Exact test, and continuous outcomes will be compared using student’s T-test for normally distributed variables, or Wilcoxon rank sum test otherwise.

Missing data will be summarized for each outcome, including the number and proportion of participants with missing observations. Analyses will primarily use available data, with denominators clearly reported for each outcome. No imputation is planned given the feasibility nature of the study.

Race/ethnicity will be a variable in descriptive analyses. These analyses are aimed at advancing equity, identifying disparities, and improving outcomes for disproportionately affected groups. Community partners support the collection and analysis of these data, including self-reported Indigenous identity. Indigenous collaborators will provide ongoing consultation and oversight of Indigenous data analysis. Findings will be interpreted in the context of historical and systemic factors that influence racial disparities, and presented with a strength-based approach [70] with the goal of benefitting racialized and Indigenous communities.

To determine if a multi-centre trial is warranted, outcomes will be interpreted based on traffic light progression criteria [78–80]. We will consider the study “green” (proceed with multi-centre trial) if the primary feasibility outcome and at least one of the secondary feasibility outcomes are met, “amber” (proceed to multi-centre trial with modifications) if only secondary feasibility outcomes are met, and “red” (do not proceed to multi-centre trial) if none of the primary or secondary feasibility outcomes are met.

### Data management plan

A comprehensive data management plan was developed using DMP Assistant (dmp-pgd.ca/). Study data are collected by research personnel via McMaster University REDCap electronic data capture tool. Only the research coordinator and principal investigator have access to the study keys. De-identified data are exported into SPSS for statistical analysis and converted to CSV files for long-term storage. Paper consent forms are stored in a locked room in a locked office for the duration of the study and confidentially shredded after the study ends. Identifiable data will be destroyed shortly after the study period concludes.

The data management plan integrates principles of Indigenous data sovereignty such as OCAP® (Ownership, Control, Access, and Possession) for First Nations. De dwa da dehs nye > s Aboriginal Health Centre has assumed responsibility for the data of Indigenous participants, with delegation of data possession and security to the research team.

### Ethics

The study protocol, consent forms, and all relevant study documentation have been approved by the Hamilton Integrated Research Ethics Board (HiREB #17708; final approval on June 2, 2025). The study will be conducted in accordance with the Canadian Tri-Council Policy Statement 2 guidelines and the 2024 revision of the Declaration of Helsinki. Any major protocol modifications will be reviewed with collaborators and submitted for Ethics approval.

### Study status

Enrollment is estimated to be complete by October 2026. Data collection is estimated to be complete by April 2027, and results available by September 2027. The original approved study protocol (Version 3, 19 Apr 2025) can be found in S2 File, with amendments as follows:

1. Version 4.0 (9 Jul 2025): Changed compensation structure for MCH staff and community co-researchers to ensure appropriate remuneration, and added a co-investigator.
2. Version 5.0 (27 Nov 2025): Extended duration of retrospective chart review from 1 year prior to study start to 3 years prior to study start, to capture data during the 2023 wave of syphilis in Hamilton that prompted this study.
3. Version 6.0 (19 May 2026): Extended study duration from 12 months to 18 months to meet enrollment target (given unexpectedly low patient volumes at MCH in 2026), and made budget adjustments based on other funding obtained.

## Discussion

Rising rates of syphilis in pregnancy and congenital syphilis demand urgent action, while centering the needs of individuals and communities most affected. This project evaluates one strategy to improve timely diagnosis and treatment of syphilis in pregnancy: implementation of HIV/syphilis POCT in a low-barrier prenatal clinic for pregnant people at risk of syphilis. A recent population-based retrospective cohort study in Ontario, Canada found that 21% of pregnancies do not receive first-trimester syphilis screening [81], highlighting the need for accessible syphilis screening options. There is a recognized research gap regarding syphilis POCT for prenatal screening in high-income countries [46]. Our study will be the first to evaluate syphilis POCT feasibility in an outpatient prenatal care setting in a high-income country. The study will also generate preliminary data on POCT effectiveness for expediting syphilis management in marginalized pregnant people and their sexual partners; partner management is an essential yet poorly addressed element of congenital syphilis prevention.

Collaboration with community partners has ensured prioritization of participant and community needs and benefits throughout the project. The study design incorporates mechanisms to promote inclusion and representativeness of the study sample, and trauma-informed research principles and cultural safety have been integrated into study processes. Concurrent community knowledge mobilization initiatives are increasing community awareness of syphilis, congenital syphilis, and accessible syphilis testing. These efforts are co-led by the YWCA Hamilton and community co-researchers, and have included creation of a pamphlet and video, tabling at community events, and arts-based workshops about syphilis at shelters and drop-in spaces for women and gender-diverse people. Art from the workshops will be collated into a zine to be distributed to community members, healthcare workers, and policymakers, in order to deepen understanding of community perspectives about syphilis. We are also building capacity for HIV/syphilis POCT by offering training and start-up packages to healthcare providers caring for populations at risk of syphilis, particularly females of child-bearing age and pregnant people. Indigenous partners are guiding knowledge mobilization efforts for their healthcare and community spaces.

Limitations of this pilot study include small sample size, which reduces statistical power and may affect sample diversity and representativeness. The unique prenatal care setting at MCH may affect generalizability of study findings; for example, the salaried pay structure at MCH may facilitate implementation of syphilis POCT more than a fee-for-service clinic model. MCH provider perspectives on the value of POCT implementation will be addressed in a separate mixed-methods study.

Results from this pilot study will inform design of a multicenter implementation trial, to generate robust data on the feasibility and effectiveness of prenatal screening with syphilis POCT in high-income countries. These data will be used to guide clinical practice and public health policy, with the ultimate aim of protecting communities from syphilis.

## Supporting information

S1 File SPIRIT 2025 checklist. (DOCX)

S2 File Approved study protocol. (PDF)
