# Supplementary material for: PREnatal Syphilis point-of-care ScreenING in an urban low-barrier clinic (PRESSING) study: Protocol for a prospective single arm feasibility trial
Source: PLoS One. 2026 Oct 22;21(10):e0360150. doi: 10.1371/journal.pone.0360150 (PMC13690238; doi:10.1371/journal.pone.0360150)
Supplement: Supplementary file 1 [file pone.0360150.s002.pdf]

PROTOCOL NAME:

PREnatal Syphilis point-of-care ScreenING in an urban low-barrier clinic (PRESSING): A prospective single arm feasibility trial

PRINCIPAL INVESTIGATOR:

Laura Erdman
Division of Pediatric Infectious Disease, Department of Pediatrics, McMaster University
McMaster Children's Hospital
1280 Main Street West, Hamilton, ON, L8S 4K1
phone: 905.521.2100 x77577
erdmanlk@mcmaster.ca

Protocol Co-Investigators:
Suzanne Turner, Department of Family Medicine, McMaster University
Mary Vaccaro, Department of Social Work, McMaster University
Medora Uppal, YWCA Hamilton
Janet Gasparelli, De Dwa Da Dehs Nye>s Aboriginal Health Centre
Jeffrey Pernica, Department of Pediatrics, McMaster University
Jacqueline Wong, Department of Pediatrics, McMaster University
Donald Brody Duncan, Department of Pathology and Molecular Medicine, McMaster University
Marek Smieja, Department of Pathology and Molecular Medicine, McMaster University
Amanda Ramdyal, Department of Family Medicine, McMaster University
Robin Lennox, Department of Family Medicine, McMaster University
Tim O'Shea, Department of Medicine, McMaster University
Anna Cvetkovic, Department of Medicine, McMaster University
Lawrence Mbuagbaw, Department of Health Research Methods, Evidence, and Impact

Protocol Contributors:
Shannon Murphy and Monique Lavallee, Niswi Kwe Consulting Group

Funded by: CIHR Operating Grant: Addressing Infectious and Congenital Syphilis in Canada (AS1-192615).

Clinical trial registration: Clinicaltrials.gov - NCT06829602

Current Protocol Version: 3.0

Study Summary

**Title**	PREnatal Syphilis point-of-care ScreenING in an urban low-barrier clinic (PRESSING): A prospective single arm feasibility trial
**Short Title**	Syphilis point-of-care screening in pregnancy
**Protocol Number**	V3.0
**Methodology**	Prospective single arm feasibility trial<br>Adjunctive prospective and retrospective chart review
**Study Duration**	1 year
**Study Center(s)**	Maternity Centre of Hamilton
**Objectives**	To implement a Health Canada-approved HIV/syphilis point-of-care test (POCT) in a low-barrier prenatal care clinic, and evaluate acceptability, staff performance, and effectiveness for expediting treatment of pregnant people with syphilis and their sexual partners.
**Number of Subjects**	1. Pregnant clients: N=62<br>2. Sexual partners of syphilis-positive pregnant clients: N=15
**Main Inclusion Criteria**	1. Pregnant clients for whom syphilis screening is indicated at a prenatal visit, and have at least one of the following risk factors for syphilis infection:<br>a. Substance use or accessed addiction services in the past 1 year<br>b. Unstable housing in the past 1 year<br>c. Multiple sexual partners in the past 1 year<br>d. History of a sexually transmitted or blood-borne infection (STBBI)<br>e. Late to prenatal care (initial prenatal visit >20 weeks).<br>2. Sexual partners of syphilis-positive pregnant clients who are physically present in the clinic.<br><br>Exclusion criteria are previous positive syphilis testing, and not competent to consent to study participation.

**Outcomes and Statistical Methodology**	The primary outcome is the proportion of pregnant participants with syphilis risk factors who undergo POCT the first time it is offered. Secondary outcomes include feasibility outcomes (proportion of pregnant participants who undergo POCT at least once during pregnancy; proportion of participants willing to undergo POCT in future; proportion of valid POCT on clinicians' first attempt; proportion of correct test interpretations by clinicians) and exploratory clinical outcomes (proportion of syphilis POCT-positive pregnant participants who undergo same-day treatment initiation; time to treatment initiation for syphilis-positive pregnant participants and partners; proportion of syphilis-positive pregnant participants and partners who receive any treatment during the pregnancy).<br><br>Descriptive statistics will be used to analyze baseline characteristics and outcomes: counts and proportions for categorical variables, and means with standard deviations or medians with ranges for continuous variables, with 95% confidence intervals as needed. Treatment-related outcomes will be compared between syphilis-positive pregnant participants diagnosed by POCT versus laboratory-based syphilis testing: Chi-square test or Fisher's Exact test for categorical variables, and T-test or Wilcoxon rank sum test for continuous variables.

1. Background and Rationale

Syphilis is a sexually transmitted and blood borne infection (STBBI) that can cause a wide spectrum of disease in adults, from asymptomatic infection to severe neurological and cardiac complications.[1] Rates of infectious syphilis in Canada have increased 3.3-fold from 2016 to 2022.[2] Females have been disproportionately affected, with rates rising from 1.7/100,000 in 2016 to 25.2/100,000 in 2022,[2] and predominantly affecting females of child-bearing age.[3] This poses risks for syphilis in pregnancy and associated adverse outcomes, such as miscarriage, stillbirth, premature delivery, and severe or fatal neonatal infection.[4-9] Congenital syphilis can cause devastating long-term complications including neurodevelopmental deficits and musculoskeletal deformities, [5] and has been linked to excess mortality in early childhood.[10] In 2022, 117 confirmed congenital syphilis cases were reported in Canada, a staggering 3000% increase since 2016.[2] This is certainly an underestimate, since congenital syphilis is challenging to confirm at birth and many neonates are presumptively treated.[5, 11]

Congenital syphilis is almost always preventable by timely diagnosis and treatment of syphilis in pregnancy, and concurrent treatment of sexual partners to avoid maternal re-infection.[12-14] Syphilis in adults is treated with intramuscular penicillin, and a single dose is often sufficient for early infection [15]. Treatment effectiveness is highest prior to third trimester.[12] However, barriers to prenatal care reduce access to diagnosis and treatment, stemming from the social determinants of health.[16, 17] In Canada, syphilis in pregnancy is associated with low income, substance use, housing instability, and rural and remote residence.[17-19] Poor access to prenatal care is prevalent among congenital syphilis cases in Canada [18, 20] and the United States,[6, 21, 22] with gaps at each step of the care cascade.[22, 23] Barriers include transportation, stigma, and negative interactions with staff. Efforts to reduce congenital syphilis must address these barriers and the upstream social determinants of health.

Indigenous communities in Canada are disproportionately affected by syphilis [17-19], related to intergenerational trauma and legacies of colonization. Indigenous people also face systemic barriers to treatment due to colonial policies and pervasive racism in healthcare systems.[24] Indigenous peoples are strong sovereign nations with inherent rights, including the right to make decisions about their lives and issues affecting their communities. It is critical to have Indigenous leadership and guidance when developing strategies to prevent congenital syphilis.

In Hamilton, Ontario, infectious syphilis rates in females increased 400% from 2019 to 2022, and resulted in 7 cases of confirmed early congenital syphilis between 2018 and 2022.[25] The Maternity Centre of Hamilton (MCH) is a key care provider for pregnant people at risk of syphilis. MCH is a community-based prenatal clinic affiliated with McMaster University's Department of Family Medicine, designed to serve urban populations facing barriers to prenatal care such as low income, substance use, housing instability, and newcomer status.[26] In keeping with the literature on low-barrier prenatal care,[27-31] MCH policies include self-referral, walk-in visits, embedded social services, and a client-centered approach. MCH offers a specialized Program for Substance Use in Pregnancy (PROSPR) for integrated prenatal and

substance use care, with a focus on harm reduction. MCH also has a robust relationship with YWCA Hamilton, and routinely receives referrals from the YWCA. YWCA operates a transitional housing program and drop-in space for women and non-binary people with experiences of homelessness, and a supervised substance consumption program. YWCA clients often have mental health concerns, substance use, experiences of gender-based violence, and/or engagement in sex work, with associated risks of unplanned pregnancy and STBBI. A YWCA reproductive health outreach worker engages pregnant clients at YWCA programs and in street-based settings, and can accompany them to clinic and support their care.

Recent informal review at MCH indicated rising numbers of syphilis in pregnancy, and a high rate in people using drugs (approximately 25%). Despite MCH's low-barrier model, 25% of syphilis cases had very delayed treatment, and 25% received no treatment during pregnancy. A major challenge is syphilis diagnostics. The conventional syphilis serology test is performed on a venous blood sample at the Public Health laboratory. It detects anti-syphilis antibodies (treponemal) and antibodies that measure disease activity (non-treponemal, or RPR). Turn-around time for a positive result is up to 6 days,[32] and in the MCH experience, 1-2 weeks. Given this timing, it may be difficult to communicate a positive result due to clients' lack of phone and internet access, or change in location due to unstable housing. Even if the result is conveyed, many barriers impede return for treatment. Clients with a history of intravenous drug use and scarred veins may have delayed or absent syphilis screening in pregnancy due to unsuccessful venipuncture. These diagnostic issues create inequitable gaps in syphilis care, with potential for adverse maternal and neonatal outcomes.

Rapid syphilis point-of-care tests (POCT) may help address these concerns.[33, 34] Syphilis POCTs are rapid immunoassays that detect treponemal antibodies in blood, and a finger-prick sample can be used. Compared to laboratory-based serological assays, POCTs have excellent specificity, and sensitivity is high in higher burden infections (RPR≥1:8).[35-37] Syphilis POCTs have been implemented in low- and middle-income countries (LMICs) given barriers to prenatal follow up and limited laboratory facilities. In these settings, POCTs are highly acceptable for pregnant clients and staff, and increase syphilis screening rates, same-day treatment, and partner treatment.[38, 39] Two cluster-randomized controlled trials comparing syphilis POCT to serological testing found conflicting effects on adverse neonatal outcomes,[40-42] though both trials had unclear to high risk of bias.[43] Modelling studies suggest that syphilis POCTs reduce adverse outcomes and are cost-effective,[44-46] even in low prevalence settings.[47] The World Health Organization endorses HIV/syphilis POCTs for antenatal screening in LMICs.[48]

Despite these positive findings, antenatal screening with syphilis POCT can present challenges [39, 49]. Some pregnant clients are hesitant to accept POCT due to fear of a positive result, or if they receive inadequate counselling about syphilis in pregnancy. System barriers include insufficient staff training and time, inadequate quality assurance, and POCT shortages.

Several prospective field studies in high-income countries (HIC) have evaluated syphilis POCT among adult (non-pregnant) outpatients at risk of syphilis.[50-55] POCT is generally acceptable [54, 55] and facilitates treatment. Among men screened by syphilis POCT in an Emergency Department, 55% of positive cases received same-day treatment.[51] Men who have sex with men who tested positive on POCT at outreach sites were all treated within 1 week.[50] In a multi-site study in Alberta (including Emergency Departments, sexual health clinics, a correctional facility, and a First Nations community), the INSTI multiplex HIV/syphilis POCT had 97.9% sensitivity (when RPR≥1:8) and 99.8% specificity. POCT facilitated same-day treatment in 85% of infectious syphilis cases.[52] This assay is now Health Canada-approved.

Syphilis POCT may be a valuable tool for expediting diagnosis and treatment of syphilis in pregnant people facing barriers to care in HICs, given the opportunity for same-day treatment initiation and use of finger-prick blood sample. To our knowledge, there are no published data evaluating syphilis POCT in this population. Acceptability of POCT and rates of same-day treatment initiation may differ from other studied groups, due to competing prenatal care priorities, withdrawal or intoxication in pregnant people using drugs, and experiences of trauma. It is also important to assess clinician performance of POCT in a busy clinical environment, and whether POCT can facilitate management of sexual partners.

We have designed a study to evaluate the feasibility of implementing the INSTI HIV/syphilis POCT for MCH pregnant clients at risk of syphilis. If promising, the results will inform design of a multicenter implementation trial to guide public health policy.

1.1 Community engagement

This study represents a collaboration between academic researchers, people with lived experience, and community organizations – YWCA Hamilton and De Dwa Da Dehs Nye>s Aboriginal Health Centre (DAHC). Community partners have co-developed study procedures and knowledge mobilization strategies, with attention to the psychological and cultural safety of participants and relevance of study results. This involvement will continue throughout all phases of the project.

1.1.1 People with lived experience

Three community members with lived experience of syphilis in pregnancy and/or substance use in pregnancy have graciously collaborated on this study. Importantly, they supported the study concept and feel that the study will benefit pregnant people affected by syphilis and/or substance use. They have reviewed study procedures and the content and wording of study materials, to improve feasibility and safety for participants.

1.1.2. YWCA Hamilton

As described above, YWCA Hamilton has extensive experience supporting people with substance use and underhousing, and understands the range of needs and perspectives among

these populations. YWCA staff have been and will continue to be intimately involved in study conception and design, particularly with regards to knowledge mobilization.

1.1.3. Indigenous communities

Syphilis is a highly stigmatized infection that disproportionately affects Indigenous communities, which is directly related to colonization, historical trauma, and ongoing racism, including in healthcare systems. Moreover, research has historically harmed Indigenous people {Hyett, S., 2018}. It is critical to engage Indigenous communities in this work through respectful relationships, to ensure benefit and minimize harm.

This project aligns with Example 3 in Section 9.2 of the Tri-Council Policy Statement: Ethical Conduct for Research Involving Humans (TCPS2): *"Research focusing on a larger community that is known to include Indigenous people (regardless of their proportion), and where Indigenous-specific conclusions are anticipated."* An estimated 10% of MCH clients self-identify as Indigenous. We acknowledge that research participation may feel unsafe or cause harm to Indigenous individuals. Additionally, while race/ethnicity-based data analysis can be a powerful tool to identify and address inequities, such analysis must be supported by the community and be framed with a strengths-based lens to avoid stigmatization.

Engagement with urban Indigenous communities is complex, given the diversity of nations represented, and no overarching governing body. TCPS2 discusses partnering with organizations like friendship centres or health access centres to facilitate ethical engagement with the communities they serve. Through an established connection with the YWCA, we approached DAHC, which provides low-barrier health care services to urban Indigenous communities in Hamilton and surrounding regions. We share common goals in supporting people affected by the social determinants of health and preventing syphilis in pregnancy and congenital syphilis. In June 2024 during the early stages of the project, we met with DAHC's Chief Executive Officer and Clinic Services Manager, who were supportive of the study. We have since developed a relationship centered on respect and reciprocity. Through a series of consultations, DAHC staff have provided guidance around cultural safety for research participants, race/ethnicity-based analysis, Indigenous data sovereignty, and knowledge mobilization. The DAHC Board of Directors requested to review the study protocol once submitted to REB. In March 2025, they reviewed the provisionally approved study protocol and a research application detailing the terms of the proposed collaboration (see Supporting Documents). Approval for the collaboration was granted. DAHC will continue to provide culturally appropriate guidance for the study; assume responsibility for the security of Indigenous data and control how these data are analyzed and presented; facilitate broader community engagement in a safe and ethical manner (e.g. currently exploring engagement of DAHC's Patient Advisory Circle); and undertake knowledge mobilization within Indigenous communities according to Indigenous ways of knowing. We approach this partnership with

humility and commitment. We will compensate DAHC for their work, provide quarterly reports on study progress, and reciprocally offer support for DAHC's mandate as they see fit.

We have also engaged Niswi Kwe, an independent Indigenous consulting group, for complementary guidance around cultural safety, race/ethnicity-based analysis, and approaches to community consultation. We are open to other forms of engagement with urban Indigenous communities as the need and opportunity arise.

2. Study Objectives

The primary objective is to measure uptake of HIV/syphilis POCT among MCH pregnant clients with pre-defined risk factors for syphilis. The primary outcome is the proportion who undergo POCT the first time it is offered. POCT uptake is an important client-centered outcome, which is critical for evaluating feasibility of implementation and informing design of a multi-site trial. We hypothesize that POCT will be acceptable, based on syphilis POCT uptake in antenatal care in LMICs [38] and HIV and Hepatitis C POCT uptake in North American populations [56-59].

The secondary objectives are:
1. To evaluate clinician performance of POCT in the clinical environment.
2. To establish baseline data regarding timeliness and adequacy of treatment of syphilis in pregnancy at MCH.
3. To evaluate whether POCT expedites treatment of syphilis-positive pregnant clients and their sexual partners.

3. Methodology

The study is primarily a prospective single arm feasibility trial, with integrated elements of chart review methodology given ethical and practical constraints (Figure 1).

Fig. 1. Study methodology

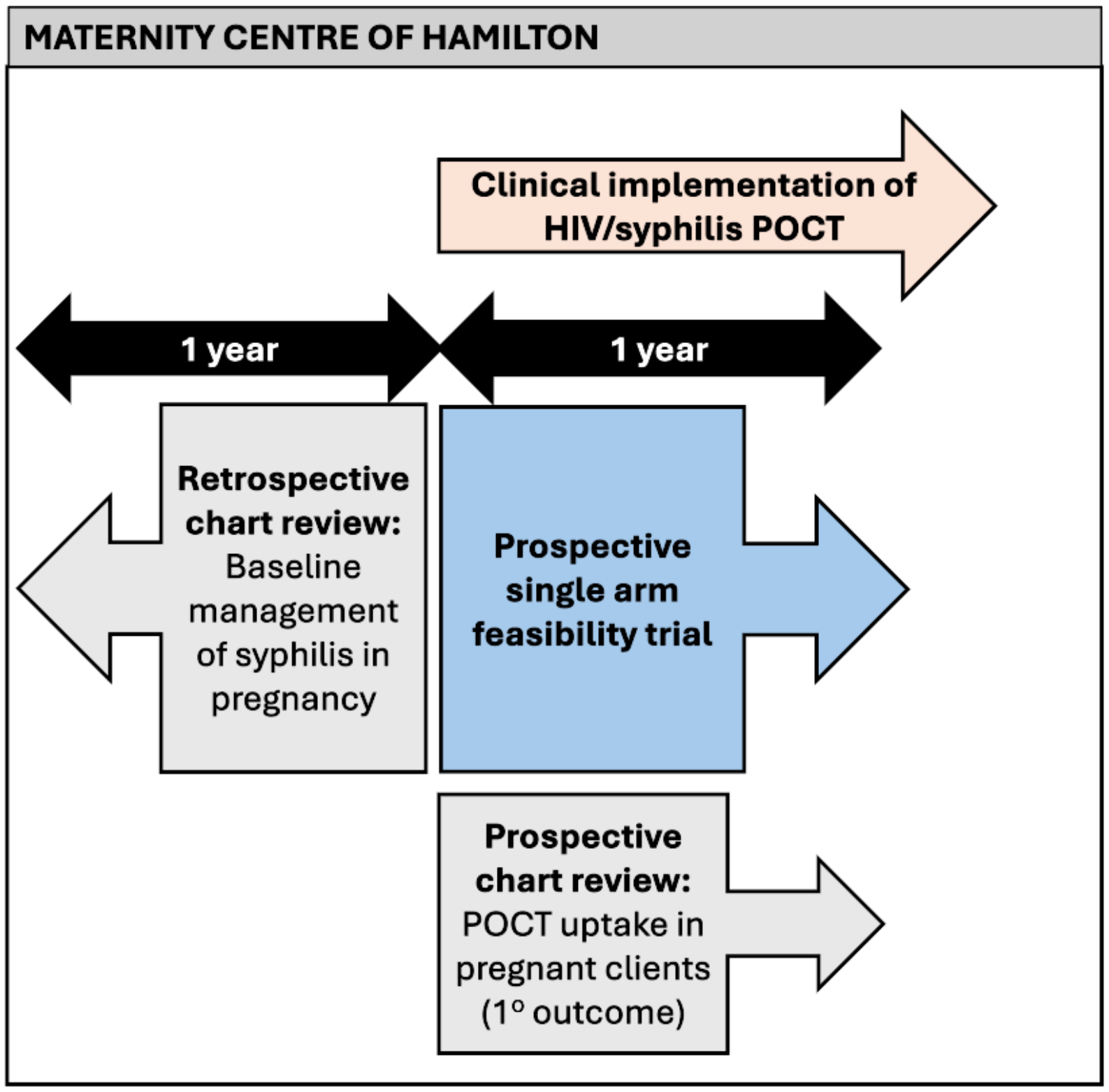

The prospective single arm feasibility trial will occur over 1 year at MCH. The Health Canada-approved INSTI Multiplex HIV-1/2 syphilis Ab test [60] will be implemented into routine clinical care for pregnant clients with pre-defined syphilis risk factors, and for the sexual partners of syphilis-positive clients. Pregnant clients and sexual partners who are offered POCT on a clinical basis will be eligible for study recruitment, and data will be collected to measure outcomes. We did not want POCT access to be contingent on research participation, as this may discourage POCT uptake for some clients. People who use substances may be less likely to engage in research, for example due to acute withdrawal. Moreover, there are barriers to research participation by Indigenous people, including historical research atrocities [61]. Declining POCT due to reluctance to participate in research could delay or prevent syphilis diagnosis during pregnancy, with potential maternal and fetal health consequences.

The prospective nature of this trial will enable robust data collection. However, we expect that a substantial proportion of MCH clients will decline study enrollment, for the reasons outlined above. This may introduce selection bias into the study population, with underrepresentation of groups who are disproportionately affected by syphilis. The primary outcome (POCT uptake) is particularly critical for understanding POCT acceptability and the feasibility of pursuing a multi-site trial. Thus, the primary outcome will be collected by prospective chart review with waived consent. To protect participant privacy, no identifying information, demographic data,

syphilis risk factors, or clinical outcomes will be collected alongside the primary outcome. Data will be aggregated, anonymized, and securely stored. While we acknowledge the importance of participant autonomy, this approach balances ethical considerations with the responsibility to generate valid, impactful research findings that can improve health outcomes.

We felt it would be unethical to include a control group with delayed access to POCT for comparison of treatment-related outcomes, given the importance of early syphilis treatment in pregnancy and risk of loss to follow up among MCH clients. Thus, we will perform a retrospective chart review at MCH to establish baseline data regarding timeliness and adequacy of treatment of syphilis in pregnancy over the 1 year prior to POCT implementation.

3.1 Study Setting

The study will be conducted at MCH, which is in downtown Hamilton and affiliated with the McMaster Family Health Team. (We have obtained permission from the McMaster Family Health Team Health Services Operations Committee to conduct this research.) MCH provides care for approximately 700 prenatal clients per year. Clients are followed by physicians and nurse practitioners – henceforth termed “clinicians.” Full-day clinics run Monday to Friday. PROSPR runs one half-day per week and follows approximately 50 clients per year for integrated prenatal and substance use care. Embedded within PROSPR is a reproductive health worker funded by the YWCA, who supports pregnant clients experiencing homelessness or substance use disorder, by facilitating transportation, accompanying clients to appointments, locating clients in the community if they have missed appointments, and addressing barriers to care. Most MCH clients given birth at the nearby hospital St Joseph’s Healthcare Hamilton.

3.2 Study Population

There are 2 study populations:

1. Pregnant clients at MCH with pre-defined risk factors for syphilis.
2. Sexual partners of syphilis-positive pregnant clients.

As part of routine clinical care, MCH will introduce HIV/syphilis POCT into the clinic for pregnant clients with risk factors for syphilis. We pragmatically defined the clinical criteria for offering POCT: among syphilis risk factors identified in Canadian literature and guidelines [15, 17, 18, 23], we selected those routinely elicited and documented during MCH prenatal assessments. (For example, history of incarceration is associated with syphilis but is not routinely discussed by MCH clinicians, and introducing this question would be stigmatizing.) To simplify study procedures and ensure real-world applicability, study inclusion and exclusion criteria were designed to be identical to clinical criteria for offering POCT.

Sexual partners of syphilis-positive clients comprise a second study population. At MCH, empiric treatment is routinely recommended for all partners of syphilis-positive pregnant clients while awaiting the partner’s serology results, to prevent re-infection of the pregnant person. In our

experience, partners may decline treatment without evidence of their own positive syphilis results. Thus, offering partners POCT may facilitate their treatment.

3.2.1. Pregnant clients with syphilis risk factors

Inclusion criteria

1. Pregnant clients at MCH with at least one of the following risk factors for syphilis, as identified by MCH clinicians:
    a. Substance use (i.e., problematic alcohol or cannabis use, non-prescribed opioid use, cocaine use, methamphetamine use, intravenous drug use) or accessing addiction services in the past 1 year;
    b. Unstable housing (i.e., homeless, street-involved, shelter use, staying with friends/family, facing eviction) in the past 1 year;
    c. Multiple sexual partners in the past 1 year;
    d. History of STBBI, as per client's report and/or medical records (HIV, Hepatitis C, Hepatitis B, gonorrhea, chlamydia, genital herpes simplex, trichomonas); or
    e. Late to prenatal care (defined by clinician consensus as initial prenatal visit >20 weeks, either at MCH or elsewhere).

AND

2. Are eligible for syphilis screening at that visit, as per Public Health guidelines:
    a. First prenatal visit;
    b. Repeat syphilis screening at 28-32 weeks if ongoing risk of syphilis infection (substance use, unstable housing, multiple sexual partners, STI diagnosis);
    c. Additional screening at clinician's discretion if ongoing risk of syphilis infection; or
    d. No prior syphilis screening.

Exclusion criteria:

1. Previous diagnosis of syphilis (by verbal report and/or chart review). POCT cannot distinguish between active syphilis and syphilis that has been adequately treated. In such cases, the non-treponemal test in laboratory-based syphilis serology is important for determining the need for treatment; or
2. Deemed to lack capacity to consent to study participation (e.g., intoxicated).

3.2.2 Sexual partners of syphilis-positive pregnant clients

Inclusion criteria:

1. Sexual partners of pregnant clients who are newly diagnosed with syphilis in pregnancy, by POCT and/or laboratory-based syphilis serology; and
2. Physically present in MCH clinic.

Exclusion criteria:
1. Previous diagnosis of syphilis (by verbal report and/or chart review); or
2. Deemed to lack capacity to consent to study participation (e.g., intoxicated).

3.3 Study procedures
Once POCT has been introduced into routine clinical practice at MCH, study procedures will consist of data collection regarding acceptability, staff performance, and effectiveness for promoting syphilis treatment of pregnant clients and their sexual partners. Figure 2 illustrates and distinguishes between routine clinical procedures and study procedures.

Fig. 2. Clinical and study procedures.

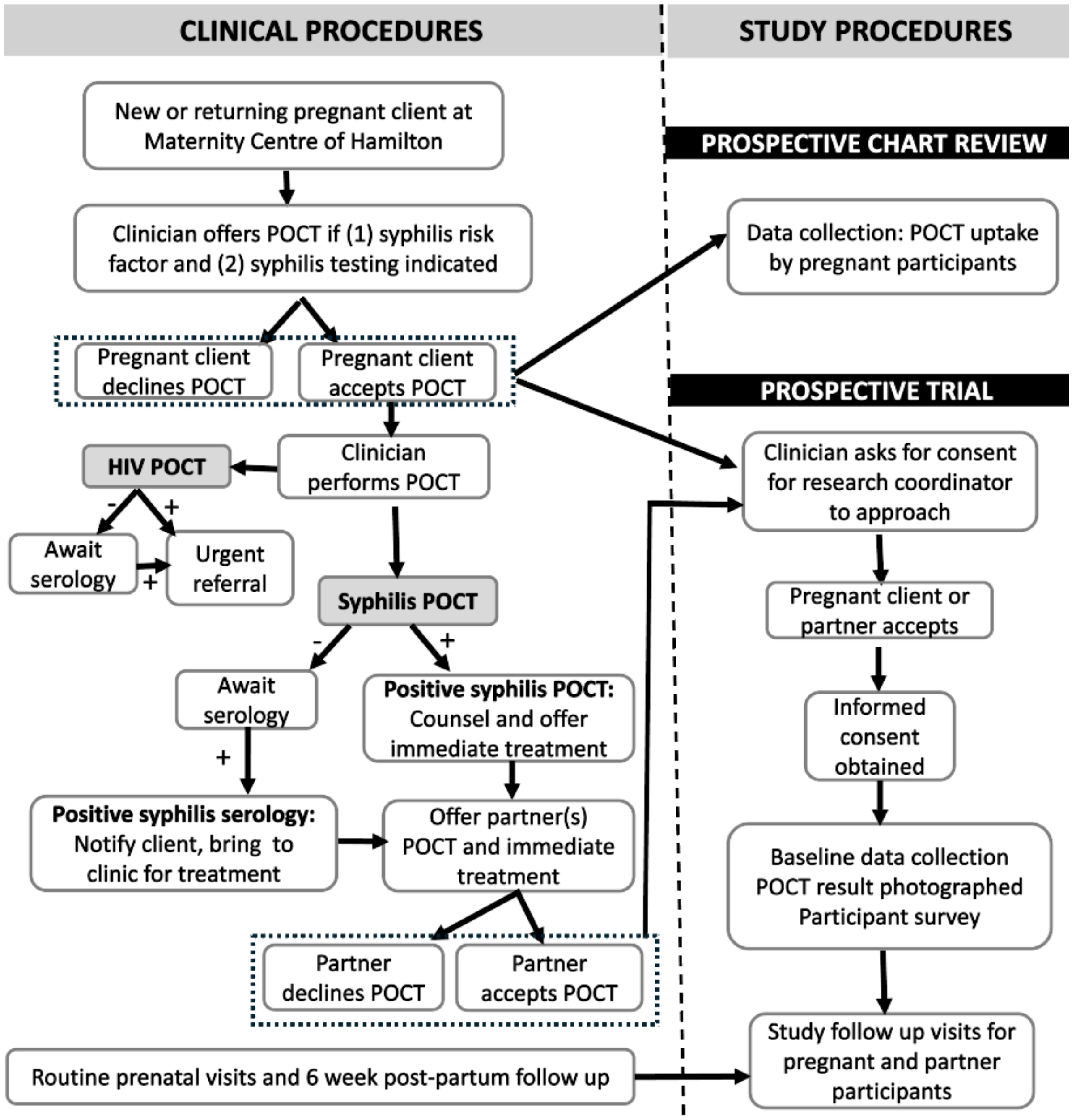

3.3.1 Routine clinical procedures

Clinicians will be trained to perform POCT assays, and a quality assurance program will be implemented. Clinicians will offer POCT to all pregnant clients with pre-defined risk factors for syphilis and no prior history of syphilis – consistent with study inclusion and exclusion criteria. Clinicians will provide standardized counselling regarding POCT and management of results. If

the syphilis POCT is positive, this will be managed as per routine practice for a positive syphilis serology. Partners will also be offered POCT and laboratory-based serologies to explore their status. Positive POCT results will be reported to Public Health as preliminary positive.

3.3.2 Study procedures

3.3.2.1 Pregnant clients

After pregnant clients are offered POCT, the clinician will introduce the study and ask if they can be approached for a consent discussion. Clients who accept and decline POCT are both eligible for recruitment. If POCT is accepted, the study will be introduced after finger-prick sample and prior to disclosure of results, since a positive POCT result may be too distressing in engage in a consent discussion. If the client agrees, the research coordinator will explain the study and informed consent. The research coordinator will be on-site during all MCH clinics. If the research coordinator is not available, another trained research staff member will fulfill this role (“research personnel”), or research personnel will call the client to arrange an in-person consent discussion another time.

Written informed consent will be obtained. There will also be an option for verbal informed consent, given expected literacy and language barriers in the study population, and consistent with other Canadian studies evaluating syphilis POCT [52, 53]. Research personnel will review both consent options with eligible participants, who can determine which option is more suitable for their circumstances. Language interpretation services will be accessed by phone.

If a pregnant client consents to study participation, research personnel will complete the enrollment form (Data Collection Form 1A, DCF-1A). The following data will be collected: age, self-reported gender and race/ethnicity, migration in the past 5 years, gestational age, pregnancy complications, substance use, housing situation, sexual partners, STBBI history, gestational age at first prenatal visit, syphilis symptoms, previous syphilis testing, whether POCT completed (reasons for declining, if given), POCT process and results, and syphilis management if POCT positive (staging, treatment, partner management). To avoid repetition and reduce stigmatization associated with sensitive questions, research personnel will obtain as much data as possible from the clinician and chart, before asking questions of the participant. The POCT result will be photographed and uploaded to a secure server, to assess diagnostic accuracy of clinicians. Research personnel will verbally administer a 2-question survey to pregnant participants about their POCT experience, or why they declined it. We restricted survey length given competing priorities in clinic, and to enable verbal administration.

Research personnel will follow up pregnant participants at every subsequent clinic visit that they are offered POCT. The following data will be collected at each follow-up (DCF-1B): updates on pregnancy complications; updates on syphilis risk factors, symptoms, testing, and management; and syphilis testing and treatment at the current follow-up. Research personnel will conduct a final follow-up with pregnant participants at the 6-week post-partum visit and

complete the final follow-up form (DCF-1C; same data as DCF-1B). If participants do not attend the scheduled follow-ups and attempts to contact them by research personnel and YWCA reproductive health worker are unsuccessful, research personnel will review the participant's MCH medical records to obtain data. (Perinatal admission records are routinely copied to MCH as the prenatal provider.)

If a pregnant client who was offered POCT declines approach by research personnel, or declines study participation, prospective chart review will be used to evaluate POCT uptake. The dates that was POCT offered, and whether POCT was completed, will be collected in DCF-2.

3.3.2.2 Sexual partners

After the sexual partner of a syphilis-positive pregnant participant is offered POCT, the clinician will introduce the study and ask if they can be approached by research personnel for a consent discussion. Partners who accept and decline POCT are eligible for recruitment, as are multiple partners. If the partner agrees, research personnel will discuss the study and the options for written or verbal informed consent, as above.

If the partner consents to study participation, research personnel will collect data in the enrollment form (DCF-3A): age, self-reported gender and race/ethnicity, migration in the past 5 years, substance use, housing situation, sexual partners, STBBI history, recent symptoms of syphilis, previous syphilis testing, whether partner underwent POCT (reasons for declining, if given), POCT process and results, and syphilis management (staging, treatment). If POCT is completed, the result will be photographed and the participant survey administered. Research personnel will follow up at the 6-week post-partum visit to complete the final follow-up form (DCF-3B). This form collects updates on syphilis risk factors, symptoms, testing, and management. If the partner does not attend the clinic visit, and attempts by research personnel to contact them are unsuccessful, research personnel will review the partner's MCH medical record to obtain data.

3.4 Outcomes

The primary outcome is the proportion of pregnant clients with syphilis risk factors who undergo POCT the first time it is offered. We specified acceptability at first offer of POCT, given the urgency of early diagnosis and treatment of syphilis in pregnancy.

Secondary outcomes consist of feasibility outcomes, which assess POCT acceptability and staff performance, and clinical outcomes, which measure POCT effectiveness for expediting syphilis treatment of pregnant and partner participants. Clinical outcomes will be exploratory, given small numbers of syphilis-positive pregnant participants. Note that "syphilis serology-positive pregnant participants" refers to positive laboratory-based syphilis serology, the reference standard for diagnosis. This definition will produce summary metrics for management of syphilis-positive cases at MCH, accounting for the breadth of social circumstances of clients,

incomplete POCT acceptance, and poor sensitivity of POCT at low RPR titers. Potential impacts of POCT will be elucidated via sub-group analysis, as described below.

Feasibility outcomes: (Feasibility thresholds in parentheses, based on desired clinical use)

1. Proportion of pregnant participants with syphilis risk factors who undergo POCT at least once during pregnancy (>80%).
2. Proportion of pregnant participants who undergo POCT, and are willing to undergo POCT in future based on post-POCT survey (>80%).
3. Proportion of partner participants who undergo POCT, and are willing to undergo POCT in future based on post-POCT survey (>80%).
4. Proportion of POCT with valid results on clinician's first attempt, as defined by the manufacturer's instructions (>90%).
5. Proportion of POCT that are correctly interpreted by the clinician, as per consensus of 2 blinded independent study staff reviewing digital photographs of the results (>90%).

Clinical outcomes:

6. Proportion of syphilis POCT-positive pregnant participants who receive same-day treatment initiation with penicillin.
7. Time to communicate test results to syphilis serology-positive pregnant participants, from date of first positive syphilis test (days).
8. Time to first penicillin treatment for syphilis serology-positive pregnant participants, from date of first positive syphilis test (days).
9. Proportion of syphilis serology-positive pregnant participants who receive any syphilis treatment in pregnancy.
10. Proportion of syphilis serology-positive pregnant participants who receive the recommended treatment in pregnancy, based on syphilis staging [15]:
    - Early latent syphilis: 1-2 weekly doses of penicillin, initiated >4 weeks prior to delivery.
    - Late latent syphilis: 3 weekly doses of penicillin, initiated >4 weeks prior to delivery.
11. Time to first penicillin treatment of partner participants, from date of pregnant person's date of first positive syphilis test (days).
12. Proportion of partner participants who receive any syphilis treatment during pregnancy.
13. Proportion of partner participants who receive the recommended syphilis treatment during pregnancy, based on their syphilis staging.

3.5 Participant Timeline

Figure 3. Schedule of Events and Follow-up

TIMEPOINT	Enrollment	Subsequent clinic visits where POCT is offered	6-week post-partum clinic visit
**PREGNANT CLIENTS**			
Eligibility screen	X		
Offer of HIV/syphilis POCT	X	X	
Informed consent	X		
Outcome assessment	X	X	X
**SEXUAL PARTNERS OF SYPHILIS-POSITIVE CLIENTS**			
Eligibility screen	X		
Offer of HIV/syphilis POCT	X		
Informed consent	X		
Outcome assessment	X		X

3.6 Sample Size Determination

We hypothesize that uptake at first offer of POCT will be 80%, based on acceptability of rapid syphilis testing in antenatal care in LMICs [38] and HIV and Hepatitis C POCT uptake in North America [56-59]. To ensure that the width of the 95% confidence interval around this estimate is at most 10%, we need to approach at least 62 pregnant clients over the 1-year study period. This number is achievable, since PROSPR clinic follows 50 clients annually (all would be eligible given substance use or accessing addictions services) and in the past year there have been at least 20 other MCH clients who fulfil inclusion criteria (e.g., past STBBI, late to prenatal care).

Based on informal review of syphilis in pregnancy at MCH, we expect 25% of the study population to be newly diagnosed with syphilis. If all clients offered POCT consent to the study, this translates to 15 newly diagnosed syphilis cases over 1 year and maximum 15 sexual partners offered POCT.

3.7 Baseline data for management of syphilis in pregnancy at MCH

To better understand the impact of POCT implementation at MCH, we will formally generate baseline data regarding management of newly diagnosed cases of syphilis in pregnancy. We will undertake a retrospective chart review over the 1 year prior to POCT implementation.

Cases will be identified by a positive syphilis serology and/or a syphilis diagnosis in the clinical issues list or Ontario Perinatal Record. Clients with previous positive syphilis serology will be excluded. Data will be abstracted by research personnel. Eligible patient records will be identified by selecting the clinic's patient roster and searching week by week through patients seen over the 1 year prior to POCT implementation (depending on study start date). Eligible patients' names will be collected to track patients and avoid duplicate data abstraction. The patient's name will be linked to a Study ID which will be stored confidentially in a separate document in a secure McMaster OneDrive file, as detailed in Section 4.2 below.

The following data will be collected in DCF-4, if available in the chart: age, self-reported gender and race/ethnicity, migration in the past 5 years, gestational age at first prenatal visit, pregnancy complications, substance use, housing situation, sexual partners, STBBI history, symptoms of syphilis, syphilis testing, and syphilis management (staging, treatment, follow up testing, and partner management).

In order to identify cases of syphilis diagnosed at delivery (which represents a missed opportunity for diagnosis that POCT may address), we will identify MCH clients without any documented syphilis serology in pregnancy. We will review their perinatal admission records, and if syphilis serology is newly positive at delivery, these charts will be extracted.

3.8 Statistical methods for primary and secondary outcomes

Descriptive statistics will be used to describe baseline characteristics of pregnant participants, syphilis-positive pregnant participants, and partner participants. Continuous variables (e.g. age) will be presented as median with first to third quartiles (Q1,Q3), or mean with standard deviation (SD) depending on the distribution. Categorical variables (e.g. self-reported gender, substance use) will be reported as counts and proportions (%). Continuous and categorical variables will be reported with 95% confidence intervals.

For primary and secondary outcomes, continuous and categorical variables will be analyzed as above. For the primary outcome, we will present descriptive analysis of demographic variables for each group (accepted POCT at first offer, or did not). Low statistical power precludes further analysis, but these preliminary data will be hypothesis-generating for future studies.

For clinical outcomes related to management of syphilis infection in pregnancy (#7-10), we will compare participants diagnosed with syphilis by POCT, versus those diagnosed by laboratory-based syphilis serology (i.e., if declined POCT, or if POCT was negative). Given risk of confounders, we will describe baseline characteristics in each group. However, since POCT-positive cases more likely reflect infectious syphilis and unstable social circumstances, earlier and/or more complete treatment in this group would argue for the value of POCT and same-day treatment initiation. Categorical outcomes will be compared using Chi-square test or Fisher's Exact test, and continuous outcomes will be compared using student's T-test for normally distributed variables, or Wilcoxon rank sum test otherwise.

Race/ethnicity (including self-reported Indigenous background) will be a variable in descriptive analyses of pregnant participants (including sub-groups divided by primary outcome and syphilis status) and partner participants. Categories will be defined according to the Canadian Institute of Health Information race-based and Indigenous identity data standards. Analyses will be aimed at advancing equity, identifying disparities, or improving outcomes for disproportionately affected groups. The distribution of race/ethnicity-based data are also important for understanding the generalizability of the study to other settings, and help to make visible ways in which vulnerable or under-represented populations liaise with the healthcare system. If there are five or less participants in any race/ethnicity category, categories will be combined in order to protect identity and privacy, or a range provided (e.g., 1-5). Given expected small numbers in each category, it will not be possible to draw robust conclusions; however, trends may be apparent. This will enable understanding of the populations accessing MCH services and affected by syphilis, and inform community-based knowledge mobilization and education around syphilis in pregnancy.

Community partners YWCA and DAHC, as well as independent Indigenous consultants Niswi Kwe support the collection and analysis of race-based data, including self-reported Indigenous background. DAHC and Niswi Kwe will provide ongoing consultation and oversight of Indigenous data analysis, to respect and uphold the principles of Indigenous data sovereignty such as OCAP® (Ownership, Control, Access, and Possession) for First Nations. There are plans to engage in broader community engagement, guided by DAHC in a safe and ethical manner. These processes will ensure that findings will be presented with a strength-based approach {Hyett, S., 2018} and benefit Indigenous communities. Any findings will be interpreted in the context of historical and systemic factors that influence racial disparities. If at any point, Indigenous partner organizations or community members are not comfortable with the Indigenous data analysis, we will follow their guidance.

Statistical tests will be performed using two-sided tests at 0.05 level of significance. P-values will be corrected for multiple comparisons. Analyses will be performed using R version 4.0.4 (R Core Team 2021) and SPSS 26 (Chicago, Illinois, USA).

3.8 Recruitment

Prospective recruitment will be carried out during all regular MCH clinics over 1 year, and will continue until the target of 62 pregnant clients offered POCT is reached.

4. Data collection and management

4.1 Retention

Research personnel will follow up with pregnant participants at subsequent clinic visits every time POCT is offered, and will follow up with both pregnant and partner participants at the 6 week post-partum clinic visit. If participants do not attend MCH at these time points, research

personnel will make multiple attempts to contact participants by phone, text, and/or email, as per the consent form. If a pregnant participant cannot be reached, the YWCA reproductive health worker will be engaged to support their access to follow-up care or connect them to research personnel for phone touch-base. Despite these efforts, we do expect some loss to follow up given the barriers faced by the study population. Obtaining consent to obtain final data from written records will mitigate this to some degree. Participants may voluntarily withdraw from the study at any time for any reason without consequence.

4.2 Data Management

Data collection is the responsibility of the research staff under the supervision of the PI. Informed consent forms will be kept on-site in a locked box in a locked cabinet that only the research team will have access to. After recruitment, research personnel will complete the relevant documentation (data collection forms) in the study database, which will be created using REDCap (Research Electronic Data Capture) software. REDCap servers are securely housed in an on-site limited access data center. All web-based information transmission is encrypted. The data are stored on a private, firewall protected network. All users are given individual user ids and passwords, and their access is restricted on a role-specific basis. REDCap was developed specifically around Health Insurance Portability and Accountability Act (HIPAA)-Security guidelines and is implemented and maintained according to McMaster University guidelines. REDCap files will be regularly backed up onto OneDrive. OneDrive is fully protected with McMaster/Microsoft security policies and only users with a McMaster ID can access it. OneDrive data is encrypted while in transit and at rest on servers. The research coordinator and PI will regularly review all data to ensure clarity and accuracy.

Some MCH clients self-identify as Indigenous, and race/ethnicity data will be collected in our study. We respect the First Nations Principles of OCAP® and the concepts of data sovereignty and information governance. The urban Indigenous community is a diverse population comprised of many nations from turtle island. DAHC has agreed to assume responsibility for the security of Indigenous data collected by the study, control how these data are analyzed and used, and ensure they are presented from a strengths-based lens. DAHC will guide further community engagement to ensure broad support for any Indigenous-specific analysis and conclusions.

5. Monitoring and Oversight

5.1. Data Monitoring Committee

Given the low-risk nature of this trial, we do not foresee the need for a data monitoring committee.

5.2. Interim Analyses

Our study will not conduct any interim analyses.

5.3. Adverse Event Reporting and Harms

We do not expect major adverse events related to study procedures. The risks of this study relate to collection of personal health and social information. Participants may experience shame or stigma when asked about sensitive topics, such as substance use or sexual history. During the consent process, we will inform participants of these upcoming questions and explain the rationale behind them – to understand who is affected by syphilis, how they perceive the test, and how we can improve their outcomes. We will collect as much information as possible from the clinician and chart before asking participants. We will emphasize that our questions are asked without judgment, and participants can decline to answer.

The personal information collected for study purposes could be subject to privacy breach. We have attempted to mitigate these risks as described elsewhere in this protocol.

6. Ethics and Dissemination

The protocol, consent forms, and all relevant study documentation will be approved by the Hamilton Integrated Research Ethics Board. Results will be disseminated as per the knowledge mobilization plan described below.

6.1. Consent and Assent

Clinicians will identify pregnant clients and sexual partners who fulfil clinical criteria for offer of POCT. The primary outcome (POCT uptake by pregnant clients) will be obtained through prospective chart review with waived consent, as we expect that a significant proportion of clients will decline study enrollment. Collection of these data (yes/no) will pose minimal risk.

The clinician will introduce the study to potential participants, and if interested, research personnel will approach them to explain the rationale for the trial, study procedures, benefits and risks of participation, and the confidential nature of participant data collection and analysis. A study handout will be provided describing syphilis in pregnancy and the purpose of the study. As compensation for their time participating in the study, participants will be offered a $20 gift card and bus fare for each study visit. Verbal or written consent will be obtained, depending on each participant's circumstances and preference. Eligible participants <16 years old will be considered mature minors and can provide informed consent without parental consent or assent required.

For retrospective chart review of syphilis-positive clients over the 1 year prior to POCT implementation, a waiver of informed consent is requested given minimal risks and expected challenges contacting participants, many of whom have precarious housing situations and inconsistent access to telecommunications. Moreover, there may be harms to contacting participants and re-visiting the details of their diagnosis with this highly stigmatized infection.

6.2. Confidentiality

All participant-related information including Data Collection Forms, photographs of POCT results, reports, etc. will be kept strictly confidential. All records will be stored in a secure, locked location that only the research staff will have access to. Participants will be assigned a unique coded number and will be identified through this number alone. The study key linking participant code number to identifying data will be stored in a separate password-protected file on a secure network. Participant data will be saved via REDCap, a securely protected and encrypted database. All members of the research team will be given a unique user ID and password and their access will be restricted on a role-specific basis. Participant records may be made available to the study sponsors, monitoring groups representative of the study sponsor, and Health Canada upon request.

6.3. Declaration of Interests

None to declare.

6.4 Ancillary and Post-study Care

Syphilis and HIV are highly stigmatized sexually transmitted infections. Discussing these infections can be difficult or triggering for clients, and receiving a positive result may be overwhelming and evoke strong emotions, such as distress, fear, shame, or anger. MCH clinicians have expertise providing non-judgmental pre-test counselling for HIV and syphilis, as well as post-diagnosis counselling, support, and management in the event of a positive result. Participants who test positive will be referred to other medical specialists as deemed clinically appropriate. They will be offered access to additional supportive resources, particularly through community partners. The YWCA reproductive health worker is available Monday to Friday and can offer individualized support on site at MCH. For Indigenous clients seeking culturally safe support, DAHC can facilitate urgent referral to their clinic.

7. Knowledge Mobilization

In order to link this research to action, knowledge mobilization strategies are under development by academics, community partners, and people with lived experience. This includes educational outreach and enhanced access to care for pregnant people and females of child-bearing age at risk of syphilis; communication of findings to the healthcare community and community organizations serving relevant populations; hands-on POCT workshops to encourage implementation in similar clinical settings; and advocacy at the policy level to fund POCT for high-risk pregnant populations. Indigenous partners will guide knowledge mobilization efforts for their health care spaces and communities, as per their judgment and understanding of community needs.

Reference List

1. Peeling, R.W., et al., *Syphilis.* Lancet, 2023. **402**(10398): p. 336-346.
2. *Infectious syphilis and congenital syphilis in Canada, 2022.* Canadian Communicable Disease Report, October 2023. **49**(10).
3. Aho, J., et al., *Rising syphilis rates in Canada, 2011-2020.* Can Commun Dis Rep, 2022. **48**(23): p. 52-60.
4. Korenromp, E.L., et al., *Global burden of maternal and congenital syphilis and associated adverse birth outcomes-Estimates for 2016 and progress since 2012.* PLoS One, 2019. **14**(2): p. e0211720.
5. Easterlin, M.C., R. Ramanathan, and T. De Beritto, *Maternal-to-Fetal Transmission of Syphilis and Congenital Syphilis.* Neoreviews, 2021. **22**(9): p. e585-e599.
6. Kimball, A., et al., *Missed Opportunities for Prevention of Congenital Syphilis - United States, 2018.* MMWR Morb Mortal Wkly Rep, 2020. **69**(22): p. 661-665.
7. Gulersen, M., et al., *Risk factors and adverse outcomes associated with syphilis infection during pregnancy.* Am J Obstet Gynecol MFM, 2023. **5**(6): p. 100957.
8. Aleem, S., et al., *Severe Congenital Syphilis in the Neonatal Intensive Care Unit: A Retrospective Case Series.* Pediatr Infect Dis J, 2022. **41**(4): p. 335-339.
9. Gomez, G.B., et al., *Untreated maternal syphilis and adverse outcomes of pregnancy: a systematic review and meta-analysis.* Bull World Health Organ, 2013. **91**: p. 217-26.
10. Paixao, E.S., et al., *Mortality in children under 5 years of age with congenital syphilis in Brazil: A nationwide cohort study.* PLoS Med, 2023. **20**(4): p. e1004209.
11. Fanella, S.B., A.; Barton, M.; Sauve, L., *Diagnosis and management of congenital syphilis – Avoiding missed opportunities*. 2024: Canadian Paediatric Society, Infectious Diseases and Immunization Committee.
12. Hawkes, S.J., G.B. Gomez, and N. Broutet, *Early antenatal care: does it make a difference to outcomes of pregnancy associated with syphilis? A systematic review and meta-analysis.* PLoS One, 2013. **8**(2): p. e56713.
13. Alexander, J.M., et al., *Efficacy of treatment for syphilis in pregnancy.* Obstet Gynecol, 1999. **93**(1): p. 5-8.
14. Blencowe, H., et al., *Lives Saved Tool supplement detection and treatment of syphilis in pregnancy to reduce syphilis related stillbirths and neonatal mortality.* BMC Public Health, 2011. **11 Suppl 3**(Suppl 3): p. S9.
15. *Public Health Agency of Canada. Syphilis guide: Key information and resources.* 5 October, 2023 5 November, 2023.]; Available from: https://www.canada.ca/en/public-

health/services/infectious-diseases/sexual-health-sexually-transmitted-infections/canadian-guidelines/syphilis.html.

16. Thean, L., A. Moore, and C. Nourse, *New trends in congenital syphilis: epidemiology, testing in pregnancy, and management.* Curr Opin Infect Dis, 2022. **35**(5): p. 452-460.
17. *Syphilis in Canada: Technical report on epidemiological trends, determinants and interventions.* 2020, Public Health Agency of Canada.
18. Gratrix, J., et al., *Outcomes of infectious syphilis in pregnant patients and maternal factors associated with congenital syphilis diagnosis, Alberta, 2017-2020.* Can Commun Dis Rep, 2022. **48**(2-3): p. 61-67.
19. Willemsma, K., et al., *Characterizing female infectious syphilis cases in British Columbia to identify opportunities for optimization of care.* Can Commun Dis Rep, 2022. **48**(2-3): p. 68-75.
20. Benoit, P., et al., *Congenital syphilis re-emergence in Winnipeg, Manitoba.* Can Commun Dis Rep, 2022. **48**(2-3): p. 89-94.
21. DiOrio, D., K. Kroeger, and A. Ross, *Social Vulnerability in Congenital Syphilis Case Mothers: Qualitative Assessment of Cases in Indiana, 2014 to 2016.* Sex Transm Dis, 2018. **45**(7): p. 447-451.
22. McDonald R, O.C.K., Torrone E, et al. , *Vital Signs: Missed Opportunities for Preventing Congenital Syphilis — United States, 2022.* MMWR Morb Mortal Wkly Rep., ePub: 7 November 2023.
23. Round, J.M., et al., *Examination of Care Milestones for Preventing Congenital Syphilis Transmission Among Syphilis-Infected Pregnant Women in Alberta, Canada: 2017-2019.* Sex Transm Dis, 2022. **49**(7): p. 477-483.
24. Smylie, J. and W. Phillips-Beck, *Truth, respect and recognition: addressing barriers to Indigenous maternity care.* CMAJ, 2019. **191**(8): p. E207-E208.
25. *Medical Advisory: Infectious Syphilis Cases Rise in Females of Childbearing Age.* Sept 22, 2023., Hamilton Public Health Services, Healthy and Safe Communities.
26. *Maternity Centre of Hamilton, Department of Family Medicine, McMaster University.* 4 November, 2023]; Available from: https://mch.mcmaster.ca/.
27. Turner, S., et al., *Guideline No. 443b: Opioid Use Throughout Women's Lifespan: Opioid Use in Pregnancy and Breastfeeding.* J Obstet Gynaecol Can, 2023. **45**(11): p. 102144.
28. Goler, N.C., et al., *Substance abuse treatment linked with prenatal visits improves perinatal outcomes: a new standard.* J Perinatol, 2008. **28**(9): p. 597-603.
29. Sword, W., et al., *Integrated programs for women with substance use issues and their children: a qualitative meta-synthesis of processes and outcomes.* Harm Reduct J, 2009. **6**: p. 32.

30. Mill, J., A. Singh, and M. Taylor, *Women in the Shadows: Prenatal Care for Street-Involved Women.* Canadian Journal of Urban Research, 2012. **21**(2): p. 68-89.
31. Heaman, M.I., et al., *Barriers, motivators and facilitators related to prenatal care utilization among inner-city women in Winnipeg, Canada: a case-control study.* BMC Pregnancy Childbirth, 2014. **14**: p. 227.
32. *Public Health Ontario: Syphilis serology.* 11 Sept 2023. [cited 2023 5 Nov]; Available from: https://www.publichealthontario.ca/en/Laboratory-Services/Test-Information-Index/Syphilis-Serology.
33. Moseley, P., et al., *Resurgence of congenital syphilis: new strategies against an old foe.* Lancet Infect Dis, 2023.
34. Naidu, P. and R.S. Tsang, *Canadian Public Health Laboratory Network guidelines for the use of point-of-care tests for Treponema pallidum in Canada.* J Assoc Med Microbiol Infect Dis Can, 2022. **7**(2): p. 85-96.
35. Angel-Muller, E., et al., *Diagnostic Accuracy of Rapid Point-of-Care Tests for Detecting Active Syphilis: A Systematic Review and Meta-Analysis.* Sex Transm Dis, 2021. **48**(12): p. e202-e208.
36. Phang Romero Casas, C., et al., *Rapid diagnostic test for antenatal syphilis screening in low-income and middle-income countries: a systematic review and meta-analysis.* BMJ Open, 2018. **8**(2): p. e018132.
37. Gliddon, H.D., et al., *A systematic review and meta-analysis of studies evaluating the performance and operational characteristics of dual point-of-care tests for HIV and syphilis.* Sex Transm Infect, 2017. **93**(S4): p. S3-S15.
38. Mabey, D.C., et al., *Point-of-care tests to strengthen health systems and save newborn lives: the case of syphilis.* PLoS Med, 2012. **9**(6): p. e1001233.
39. Swartzendruber, A., et al., *Introduction of rapid syphilis testing in antenatal care: A systematic review of the impact on HIV and syphilis testing uptake and coverage.* Int J Gynaecol Obstet, 2015. **130 Suppl 1**(Suppl 1): p. S15-21.
40. Brandenburger, D. and E. Ambrosino, *The impact of antenatal syphilis point of care testing on pregnancy outcomes: A systematic review.* PLoS One, 2021. **16**(3): p. e0247649.
41. Myer, L., et al., *Impact of on-site testing for maternal syphilis on treatment delays, treatment rates, and perinatal mortality in rural South Africa: a randomised controlled trial.* Sex Transm Infect, 2003. **79**(3): p. 208-13.
42. Munkhuu, B., et al., *One-stop service for antenatal syphilis screening and prevention of congenital syphilis in Ulaanbaatar, Mongolia: a cluster randomized trial.* Sex Transm Dis, 2009. **36**(11): p. 714-20.

43. Shahrook, S., et al., *Strategies of testing for syphilis during pregnancy.* Cochrane Database Syst Rev, 2014(10): p. CD010385.
44. Kuznik, A., et al., *Antenatal syphilis screening using point-of-care testing in Sub-Saharan African countries: a cost-effectiveness analysis.* PLoS Med, 2013. **10**(11): p. e1001545.
45. Kuznik, A., et al., *Antenatal syphilis screening using point-of-care testing in low- and middle-income countries in Asia and Latin America: a cost-effectiveness analysis.* PLoS One, 2015. **10**(5): p. e0127379.
46. Saweri, O.P.M., et al., *Economic evaluation of point-of-care testing and treatment for sexually transmitted and genital infections in pregnancy in low- and middle-income countries: A systematic review.* PLoS One, 2021. **16**(6): p. e0253135.
47. Mallma, P., et al., *Rapid Syphilis Testing Is Cost-Effective Even in Low-Prevalence Settings: The CISNE-PERU Experience.* PLoS One, 2016. **11**(3): p. e0149568.
48. Organization., W.H., *Policy brief: Dual HIV/Syphilis rapid diagnostic rests can be used as the first test in antenatal care.* 2019.
49. Zhang, Y., et al., *Barriers and facilitators to HIV and syphilis rapid diagnostic testing in antenatal care settings in low-income and middle-income countries: a systematic review.* BMJ Glob Health, 2022. **7**(11).
50. Obafemi, O.A., et al., *Rapid Syphilis Testing for Men Who Have Sex With Men in Outreach Settings: Evaluation of Test Performance and Impact on Time to Treatment.* Sex Transm Dis, 2019. **46**(3): p. 191-195.
51. Fakile, Y.F., et al., *Evaluation of a Rapid Syphilis Test in an Emergency Department Setting in Detroit, Michigan.* Sex Transm Dis, 2019. **46**(7): p. 429-433.
52. Singh, A.E., et al., *Sensitivity and specificity of two investigational Point of care tests for Syphilis and HIV (PoSH Study) for the diagnosis and treatment of infectious syphilis in Canada: a cross-sectional study.* Clin Microbiol Infect, 2023. **29**(7): p. 940 e1-940 e7.
53. Caya, C., et al., *Rapid diagnostic testing for syphilis in Arctic communities (the STAR study): a multisite prospective field diagnostic accuracy study in an intended-use setting.* Clin Microbiol Infect, 2023. **29**(10): p. 1335 e1-1335 e7.
54. Sherriff, N., et al., *Independent clinic-based evaluation of dual POCTs for screening for HIV and syphilis in men who have sex with men in Italy, Malta, Peru, and the United Kingdom.* BMC Infect Dis, 2024. **24**(Suppl 1): p. 192.
55. Maliszewski, K.N., et al., *An Evaluation of the Performance, Patient Acceptability and Feasibility of a Point-of-Care HIV-Syphilis Assay in an Urban Emergency Department.* Sex Transm Dis, 2024.
56. Minichiello, A., et al., *HIV Point-of-Care Testing in Canadian Settings: A Scoping Review.* Front Public Health, 2017. **5**: p. 76.

57. Latham, N.H., et al., *Community-based, point-of-care hepatitis C testing: perspectives and preferences of people who inject drugs.* J Viral Hepat, 2019. **26**(7): p. 919-922.
58. Bajis, S., et al., *Acceptability and preferences of point-of-care finger-stick whole-blood and venepuncture hepatitis C virus testing among people who inject drugs in Australia.* Int J Drug Policy, 2018. **61**: p. 23-30.
59. Lettner, B., et al., *Rapid hepatitis C virus point-of-care RNA testing and treatment at an integrated supervised consumption service in Toronto, Canada: a prospective, observational cohort study.* Lancet Reg Health Am, 2023. **22**: p. 100490.
60. *INSTI Multiplex HIV-1 / HIV-2 / Syphilis Antibody Test* https://www.insti.com/multiplex-test/.
61. Hyett, S., S. Marjerrison, and C. Gabel, *Improving health research among Indigenous Peoples in Canada.* CMAJ, 2018. **190**(20): p. E616-E621.
